# Monitoring Indoor Exposure to Organophosphate Flame Retardants: Hand Wipes and House Dust

**DOI:** 10.1289/ehp.1408669

**Published:** 2014-10-24

**Authors:** Kate Hoffman, Stavros Garantziotis, Linda S. Birnbaum, Heather M. Stapleton

**Affiliations:** 1 Nicholas School of the Environment, Duke University, Durham, North Carolina USA; 2 National Institute of Environmental Health Sciences, National Institutes of Health (NIH), Department of Health and Human Resources (DHHS), Research Triangle Park, North Carolina USA; 3 National Cancer Institute, NIH, DHHS, Research Triangle Park, North Carolina USA

## Abstract

Background: Organophosphate flame retardants (PFRs) are becoming popular replacements for the phased-out polybrominated diphenyl ether (PBDE) mixtures, and they are now commonly detected in indoor environments. However, little is known about human exposure to PFRs because they cannot be easily measured in blood or serum.

Objectives: To investigate relationships between the home environment and internal exposure, we assessed associations between two PFRs, tris(1,3-dichloropropyl) phosphate (TDCIPP) and triphenyl phosphate (TPHP), in paired hand wipe and dust samples and concentrations of their metabolites in urine samples (*n* = 53). We also assessed short-term variation in urinary metabolite concentrations (*n* = 11 participants; *n* = 49 samples).

Methods: Adult volunteers in North Carolina, USA, completed questionnaires and provided urine, hand wipe, and household dust samples. PFRs and PBDEs were measured in hand wipes and dust, and bis(1,3-dichloropropyl) phosphate (BDCIPP) and diphenyl phosphate (DPHP), metabolites of TDCIPP and TPHP, were measured in urine.

Results: TDCIPP and TPHP were detected frequently in hand wipes and dust (> 86.8%), with geometric mean concentrations exceeding those of PBDEs. Unlike PBDEs, dust TDCIPP and TPHP levels were not associated with hand wipes. However, hand wipe levels were associated with urinary metabolites. Participants with the highest hand wipe TPHP mass, for instance, had DPHP levels 2.42 times those of participants with the lowest levels (95% CI: 1.23, 4.77). Women had higher levels of DPHP, but not BDCIPP. BDCIPP and DPHP concentrations were moderately to strongly reliable over 5 consecutive days (intraclass correlation coefficients of 0.81 and 0.51, respectively).

Conclusions: PFR exposures are widespread, and hand-to-mouth contact or dermal absorption may be important pathways of exposure.

Citation: Hoffman K, Garantziotis S, Birnbaum LS, Stapleton HM. 2015. Monitoring indoor exposure to organophosphate flame retardants: hand wipes and house dust. Environ Health Perspect 123:160–165; http://dx.doi.org/10.1289/ehp.1408669

## Introduction

Consumer products and construction materials are frequently treated with flame retardants (FRs) to reduce their flammability and meet fire safety standards. Historically, polybrominated diphenyl ethers (PBDEs) were used as the primary FRs in polyurethane foam and electronics. However, concern over the persistence, bioaccumulation, and toxicity of PBDEs led to regulatory actions and drastic reductions in their use beginning in the mid-2000s. During the same period, the use of alternative FRs increased, allowing manufacturers to maintain compliance with fire safety standards and regulations (Stapleton et al. 2012b; van der Veen and de Boer 2012). Organophosphate FRs (PFRs), such as triphenyl phosphate (TPHP) and tris(1,3-dichloropropyl) phosphate (TDCIPP), are now among the most commonly used PBDE alternatives in consumer products containing polyurethane foam (Stapleton et al. 2011, 2012b; van der Veen and de Boer 2012). In previous work, for example, we found that TDCIPP was the most commonly detected FR in polyurethane foam samples taken from both baby products (Stapleton et al. 2011) and from residential furniture purchased after 2005 (Stapleton et al. 2012b).

Like their PBDE predecessors, PFRs are added during the manufacturing process and are not chemically bound to the products in which they are used, allowing them to escape into the environment over time. TDCIPP and TPHP have been ubiquitously detected in household, office, and automobile dust samples, suggesting that the general population comes into contact with these chemicals frequently (Carignan et al. 2013; Meeker and Stapleton 2010; Stapleton et al. 2009). Our previous work examining pathways of human exposure to PBDEs indicates that exposure to contaminated dust is associated with higher body burdens, and that hand-to-mouth behaviors may be an important pathway by which PBDEs enter the body (Stapleton et al. 2012a; Watkins et al. 2011). It remains unclear whether these relationships also apply for PFRs, although correlations between the levels of TDCIPP in dust and its primary urinary metabolite [bis(1,3-dichloropropyl) phosphate (BDCIPP)] have been reported (Carignan et al. 2013; Meeker et al. 2013). In the present study, we examined relationships between TDCIPP and TPHP concentrations in the home environment and internal exposure using concurrent measures in hand wipes and household dust, and measures of their metabolites in urine [i.e., BDCIPP and diphenyl phosphate (DPHP), respectively]. In addition, we examined associations between urinary metabolite levels and demographic (e.g., age and sex) and personal habits (e.g., hand-washing behavior) to determine their potential influence on exposure. Finally, we sought to compare levels of PFRs in house dust and hand wipes to the levels of PBDEs measured in the same samples.

## Methods

*Study design*. Healthy adult volunteers were recruited from the general population to the National Institute of Environmental Health Sciences (NIEHS) Clinical Research Unit (CRU) in 2012 (*n* = 64) using study flyers and word of mouth. Eligible participants were at least 18 years of age and had never been diagnosed with a kidney problem (not including kidney stones). One group of volunteers (paired sample group; *n* = 53) was asked to complete demographic and behavioral questionnaires and provide spot urine samples at the CRU, and to collect dust samples in their homes. A second group of participants (*n* = 11) was asked to provide daily spot urine samples at the CRU on 5 consecutive days. All study protocols were approved by the NIEHS Institutional Review Board, and all participants gave informed consent prior to providing information or samples.

*Questionnaires*. Participants provided information on their personal characteristics, including age, sex, race, height, and weight, the latter two of which were used to calculate body mass index (BMI). Participants also completed a questionnaire designed to obtain information about their personal habits, such as the average number of hours spent active in the home and the average number of times participants washed their hands per day. Information on hand washing was collected as never, 1–2 times/day, 3–5 times/day, 6–8 times/day, and > 8 times/day. For analyses, we collapsed hand washing into two categories: < 8 times/day (low hand washing) and ≥ 8 times/day (frequent hand washing), with the categorizations determined based on the distribution of responses in our study population. The frequency of hand-sanitizer gel use was also obtained; participants were classified as users or never-users of hand-sanitizer gel. Response categories for the average time spent active in the home and the average time spent driving each day were also dichotomized for analyses (≤ 8 hr/day and > 8 hr/day for time active in the home; ≤ 1 hr/day and > 1 hr/day for driving time).

*Dust collection*. Each participant was provided with instructions and a kit for the collection of household dust. Participants were instructed to insert a nylon dust collection thimble into the hose attachment of their vacuum cleaner, similar to the method used in a previous study (Stapleton et al. 2012a). Then, they vacuumed the floor in the main living area of their home for exactly 2 min (over any type of flooring). The thimble was then removed from the vacuum, sealed in a plastic bag, and returned to the CRU. The nylon thimbles were never in contact with the plastic bag. Upon receipt in the laboratory, the thimbles were removed, and the dust sieved to < 500 μm and then stored in amber glass vials at room temperature until analysis (*n* = 49; 4 participants did not provide dust samples).

*Hand wipe collection*. Hand wipe samples were collected by CRU staff (wearing gloves) using previously described protocols (Stapleton et al. 2008). Briefly, for each participant a sterile gauze wipe was soaked in 3.0 mL isopropyl alcohol, and the entire surface of each participant’s hands was wiped two times from the fingers to the wrist. Wipes (*n* = 53) were first wrapped in aluminum foil and then sealed in individual plastic bags and stored at –20°C until analysis. Field blanks (*n* = 5) were also collected to examine potential background contamination in the clinic.

*Urine collection*. Study participants provided spot urine samples during visits to the CRU (visits conducted between 0830 and 1630 hours). Urine samples were collected in standard polypropylene specimen containers and were stored at –20°C until analysis. All participants in the paired-sample group provided urine samples (*n* = 53), and participants providing repeated samples contributed a total of 49 samples (from 11 participants).

*Dust and hand wipe sample processing*. Hand wipe and dust samples were extracted in the laboratory and analyzed for brominated and organophosphate FRs including BDE-47, BDE-99, BDE-100, BDE-153, BDE-154, BDE-209, TPHP, and TDCIPP. Each hand wipe sample was extracted using a Soxhlet apparatus. Prior to Soxhlet extraction, each sample was spiked with four internal standards: d_15_-TDCIPP (155 ng), d_15_-TPHP (100 ng), a monofluorinated tetrabrominated diphenyl ether (F-BDE-69; 50 ng), and ^13^C-BDE-209 (100 ng) (Stapleton et al. 2014). Laboratory blanks (three new sterile gauze pads) were prepared as described for hand wipe samples and run next to the hand wipe samples. After Soxhlet extraction, each extract was concentrated using an automated nitrogen evaporation system (Turbo Vap II, Zymark Inc.) and transferred to a 4.0 mL amber vial, stored in a –20°C freezer. Extracts were then cleaned using Florisil solid-phase extraction (Supelclean ENVI-Florisil, 6 mL, 500-mg bed weight; Supelco), eluting the F1 fraction with 10 mL hexane (PBDEs) and the F2 fraction with 10 mL ethyl acetate (PFRs), based on the method developed by Van den Eede et al. (2012). Each fraction was concentrated to approximately 1 mL using a nitrogen concentration system and transferred to an autosampler vial (ASV) for gas chromatography–mass spectrometry (GC/MS) analysis (Stapleton et al. 2014). Dust samples (~ 100 mg) were extracted with 10 mL of 50:50 dichloromethane (DCM):hexane using sonication. This process was repeated three times, and the combined extract (~ 30 mL) was concentrated using an automated nitrogen evaporation system (Turbo Vap II), transferred to a 4.0 mL amber vial, and stored at –20°C. The dust extracts were cleaned using the same method as described above for the hand wipe samples. To measure recovery of the brominated internal standards, the extracts were spiked with 2,2´,3,4,5,5´-hexachloro[^13^C_12_] diphenyl ether (^13^C-CDE 141); d_9_-TCEP was spiked into each sample to measure recovery of d_15_-TDCIPP and d_15_-TPHP. Recoveries of F-BDE-69, ^13^C-BDE-209, d_15_-TDCIPP, and d_15_-TPHP averaged 91 ± 18%, 63 ± 17%, 75 ± 11%, and 75 ± 7%, respectively, in all samples. Analysis of laboratory blanks (*n* = 5) and an indoor dust Standard Reference Material (SRM 2585; National Institute of Standards and Technology) were also performed for quality assurance and quality control. FR measurements in hand wipes were blank subtracted using the average mass of FR measured in the field blanks. Method detection limits (MDLs) were calculated using three times the SD of the appropriate blank (i.e., dust or hand wipe). MDLs for the PFRs ranged from 0.6 ng/g for TPP to 20.0 ng/g for TDCPP in dust laboratory blanks. In hand wipes, MDLs ranged from 10 to 15 ng for the PFRs. Measured PBDE levels in SRM 2585 ranged from 78 to 130% of certified values. Measurements of TPHP and TDCIPP in SRM 2585 were 520 ± 34, and 1,820 ± 90 ng/g, respectively. These values are very similar to reports published by Van den Eede et al. (2011), and Bergh et al. (2012).

*Urine processing and analysis*. Urine samples were assessed for the primary metabolites of TDCIPP and TPHP, BDCIPP and DPHP, respectively, following methods described by Cooper et al. (2011). Briefly, BDCIPP and DPHP were measured using mixed-mode anion exchange solid-phase extraction and a mass-labeled internal standard (d_10_-BDCIPP and d_10_-DPHP) with analysis by atmospheric pressure chemical ionization liquid chromatography-tandem mass spectrometry (Cooper et al. 2011). We evaluated the recovery of d_10_-BDCIPP and d_10_-DPHP in all samples, and measured BDCIPP and DPHP concentrations in laboratory blanks (*n* = 5) for quality assurance purposes. Average recoveries of d_10_-BDCIPP and d_10_-DPHP were 78 ± 20 and 82 ± 4%, respectively. Very small amounts of DPHP were detected in laboratory blanks, whereas BDCIPP was not detected. Therefore, the MDL was calculated using three times the SD of the blanks normalized to the urine volume extracted. To account for urine dilution, specific gravity (SG) was also measured in each urine sample prior to analysis using a digital handheld refractometer (Atago). Creatinine, an alternative means of adjusting for dilution, was not measured in samples, because it varies considerably by age and sex (James et al. 1988).

*Statistical analyses*. In statistical analyses, we imputed concentrations < MDL as the MDL divided by the square root of 2. For congeners that were detected in > 70% of samples, we calculated Spearman correlation coefficients (*r*_S_) to determine the associations between continuous household dust, hand wipes, and urine levels (BDCIPP and DPHP only). Our preliminary investigations indicated that concentrations of PFR, PBDE, and PFR metabolites were log-normally distributed; therefore, log_10_-transformed values were used in all other statistical analyses.

We used linear regression models to determine predictors of continuous levels of PFRs and PBDEs in hand wipes and of PFR metabolites in urine samples (continuous outcome measures were log_10_-transformed). To aid in the interpretation of results, we exponentiated beta coefficients (10^β^), producing the multiplicative change in outcome. As predictors of congener levels in hand wipes, dust concentrations were categorized into tertiles, and as predictors of urinary PFR metabolites, both dust and hand wipe concentrations were categorized to minimize the effect of skewed data and outliers in regression analyses.

As a measure of temporal reliability of BDCIPP and DPHP in urine, we calculated intraclass correlation coefficients (ICCs) and 95% confidence intervals (CIs) (Hamer 1995; Shrout and Fleiss 1979). ICCs provide a measure of the reliability of repeated measures over time and are calculated by taking the ratio of the between-subject variability to the sum of the between- and within-subject variability (Rosner 2000). In addition, to determine whether the correlations between time points deteriorated over time, we assessed Spearman correlations between each set of time points (e.g., time 1/time 2 and time 1/time 3). Statistical analyses were performed in SAS (version 9.2; SAS Institute Inc.), with statistical significance defined as α = 0.05.

To investigate the impacts of differences in urine dilution on results, we conducted analyses of urinary metabolites using raw BDCIPP and DPHP measures as well as using SG-corrected concentrations (Boeniger et al. 1993). Three participants had very dilute urine (SG < 1.005). Measured levels of BDCIPP and DPHP were nondetectable for these participants; however, accounting for urinary dilution resulted in large corrected value estimates. Because there was substantial uncertainty around these estimate concentrations, we excluded these three participants from analyses investigating the impact of SG correction. Results using each method were very similar; thus, we chose to present uncorrected analyses including all participants.

## Results

Of the 53 adults who completed demographic and behavioral questionnaires, approximately half were male (49.1%), and the majority reported white race (75.5%) and non-Hispanic ethnicity (94.3%). Participants averaged 43.6 years of age at the time of the study (range 19–67 years).

*TDCIPP and TPHP in dust*. TDCIPP and TPHP were detected in all dust samples collected in participants’ homes (Table 1). Levels of TDCIPP and TPHP were highly variable in house dust, with the highest concentrations being 200 and 400 times the lowest concentrations, respectively. BDE-47, BDE-99, BDE-100, BDE-153, BDE-154, and BDE-209 were also detected frequently in dust samples (≥ 87.5% detects for all congeners). With the exception of BDE-209, the geometric mean (GM) concentrations of TDCIPP and TPHP were greater than those of the individual PBDE congeners assessed; however, levels of TDCIPP and TPHP were comparable to the sum of the pentaBDE congeners that were used in applications similar to those of PFRs until the early 2000s [i.e., the sum of BDE-47, BDE-99, BDE-100, and BDE-153; GM pentaBDE = 1117.8 ng/g (Stapleton et al. 2009)]. TDCIPP concentrations in dust were significantly correlated with PBDE congeners in dust (*r*_S_ = 0.50–0.57; Table 2). Levels of TPHP and BDE-47, BDE-100, and BDE-209 in dust were also correlated (*r*_S_ = 0.37, 0.33, and 0.29, respectively) although the magnitudes of correlations were lower than for TDCIPP.

**Table 1:** GM and range of flame retardants in household dust (*n *= 49) and hand wipes (*n *= 53) collected from North Carolina adults.

Congener	Dust (ng/g)			Hand wipes (ng)		
	Percent detects	GM	Range	Percent detects	GM	Range
TDCIPP	100.0	1,390	197–39,530	90.6	84.1	ND–537
TPHP	100.0	1,020	99.5–40,350	86.8	62.1	ND–1,230
BDE-47	100.0	374	28.4–21,800	100.0	18.4	2.5–454
BDE-99	100.0	510	29.8–17,280	100.0	26.0	4.4–707
BDE-100	100.0	128	19.3–4,702	81.1	2.8	ND–128
BDE-153	91.7	52.2	ND–2,609	90.6	1.3	ND–67.9
BDE-154	87.5	45.5	ND–1,969	86.8	1.0	ND–59.8
BDE-209	100.0	1,280	103–44,900	96.2	19.5	ND–804
ND, not detected.						

**Table 2:** Correlation matrix for flame retardants levels measured in paired hand wipes and household dust.

		Dust								Hand wipes							
		TDCIPP	TPHP	BDE-47	BDE-99	BDE-100	BDE-153	BDE-154	BDE-209	TDCIPP	TPHP	BDE-47	BDE-99	BDE-100	BDE-153	BDE-154	BDE-209
Dust	TDCIPP	1.00
	TPHP	0.17	1.00
	BDE-47	0.50^†^	0.37^#^	1.00
	BDE-99	0.54^†^	0.22	0.90^†^	1.00
	BDE‑100	0.55^†^	0.33*	0.96^†^	0.94^†^	1.00
	BDE-153	0.57^†^	0.23	0.88^†^	0.90^†^	0.95^†^	1.00
	BDE-154	0.56^†^	0.26	0.92^†^	0.93^†^	0.98^†^	0.97^†^	1.00
	BDE-209	0.54^†^	0.29*	0.34*	0.31*	0.41^#^	0.42^#^	0.44^†^	1.00
Hand wipes	TDCIPP	0.10	–0.05	–0.07	–0.13	–0.09	–0.06	–0.06	–0.03	1.00
	TPHP	–0.09	0.18	–0.12	–0.15	–0.14	–0.21	–0.17	–0.10	0.42^#^	1.00
	BDE-47	0.17	0.15	0.38^#^	0.34*	0.38^#^	0.38^#^	0.37^#^	0.11	0.39^#^	0.32*	1.00
	BDE-99	0.27	0.23	0.47^†^	0.43*	0.46^†^	0.49^†^	0.46^†^	0.20	0.32*	0.21	0.88^†^	1.00
	BDE-100	0.11	0.12	0.40*	0.34*	0.41^#^	0.43^#^	0.41^#^	0.13	0.33*	0.22	0.89^†^	0.85^†^	1.00
	BDE-153	0.27	0.10	0.40^#^	0.38^#^	0.42^#^	0.49^†^	0.46^†^	0.20	0.40^#^	0.20	0.84^†^	0.88^†^	0.88^†^	1.00
	BDE-154	0.22	0.17	0.38^#^	0.36*	0.39^#^	0.43^#^	0.41^#^	0.22	0.35*	0.06	0.83^†^	0.85^†^	0.87^†^	0.86^†^	1.00
	BDE-209	–0.03	0.12	0.17	0.24	0.18	0.16	0.20	0.33*	0.16	0.06	0.20	0.20	0.14	0.20	0.13	1.00
Analyses were conducted using dust and hand wipe data in which the detection frequency was > 70%. Shaded correlations indicate relationships between the same congener measured in dust and hand wipes. * < 0.05. ^#^ < 0.01. ^†^ < 0.001.																	

*TDCIPP and TPHP in hand wipes*. TDCIPP and TPHP were also detected frequently in hand wipe samples (90.6% and 86.8%, respectively; Table 1). The GM concentrations of TDCIPP and TPHP on participants’ hands exceeded those of individual PBDE congeners, which were also detected in nearly all hand wipe samples. TDCIPP and TPHP were moderately correlated with each other in hand wipes (*r*_S_ = 0.42, *p* = 0.002; Table 2). The levels of TDCIPP on participants’ hands were correlated with the levels of BDE-47, BDE-99, BDE-100, BDE-153, and BDE-154 on hand wipes. Although the levels of PBDEs in house dust and hand wipes were moderately correlated (*r*_S_ = 0.33–0.49), TDCIPP and TPHP levels were not correlated between the two matrices (Table 2). We used linear regression models with categorized dust concentrations to further explore the relationship between FRs in hand wipes and dust. As in the correlation analyses, we did not observe evidence of associations between the levels of TDCIPP or TPHP on participants’ hands and the levels in household dust (Table 3). Increasing levels of PBDEs in house dust, however, were strongly associated with their levels on hand wipes. For example, participants with the highest dust levels (3rd tertile) of BDE-100 in their homes averaged 3.44 times (95% CI: 1.25, 9.44) the levels of BDE-100 in hand wipe samples compared with those with the lowest dust levels (Table 3).

**Table 3:** Regression analyses for dust congener levels as predictors of hand wipe flame retardant levels.

Flame retardant	Low dust levels	Mid dust levels		High dust levels	
		Coefficient^*a*^ (95% CI)	*p*-Value	Coefficient^*a*^ (95% CI)	*p*-Value
TDCIPP	Reference	0.90 (0.45, 1.84)	0.78	1.18 (0.59, 2.39)	0.63
TPHP	Reference	1.20 (0.51, 2.82)	0.66	1.08 (0.46, 2.54)	0.85
BDE-47	Reference	1.36 (0.55, 3.37)	0.50	2.62 (1.05, 6.49)	0.04
BDE-99	Reference	1.29 (0.56, 2.97)	0.55	2.45 (1.06, 5.66)	0.04
BDE-100	Reference	1.61 (0.59, 4.43)	0.35	3.44 (1.25, 9.44)	0.02
BDE-153	Reference	2.94 (0.99, 8.75)	0.05	5.13 (1.73, 15.22)	0.004
BDE-154	Reference	2.16 (0.73, 6.41)	0.15	3.49 (1.18, 10.35)	0.03
BDE-209	Reference	2.34 (0.90, 6.06)	0.08	2.32 (0.89, 6.01)	0.08
Analyses were conducted using dust and hand wipe data in which the detection frequency was > 70%. ^***a***^Exponentiated beta coefficients were used to represent the multiplicative change in urine concentrations relative to the reference group.					

We also used linear regression models to investigate associations of demographic and behavioral information with the levels of FRs in hand wipes. Associations were generally imprecisely estimated and did not follow a consistent pattern across FRs (see Supplemental Material, Table S1). For example, our results suggest inverse associations between hand washing frequency (< 8 times/day vs. ≥ 8 times/day) and hand wipe concentrations of TDCIPP, BDE-47, BDE-99, BDE-100, BDE-153, and BDE-154, whereas frequent hand washing tended to be related to higher TPHP and BDE-209 levels on participants’ hands.

*DPHP and BDCIPP in urine*. DPHP and BDCIPP were detected frequently (90.6% and 83.0%, respectively) in urine samples from participants with paired house dust and hand wipe samples, with GMs of 1.02 ng/mL and 0.37 ng/mL, respectively (*n* = 53 samples). Concentrations ranged from nondetectable to 9.09 ng/mL for DPHP, and from nondetectable to 4.46 ng/mL for BDCIPP. The levels of TDCIPP and TPHP in dust were not correlated with the measures of their metabolites in urine (Table 4). Spearman correlation coefficients suggested an association between the levels of TPHP in hand wipes and the levels of DPHP in urine (*r*_S_ = 0.37, *p* = 0.006; Table 4) and the levels of TDCIPP in dust and BDCIPP in urine (*r*_S_ = 0.27, *p* = 0.06; Table 4). We conducted regression analyses using categorical versions of hand wipe and house dust variables as predictors of urinary BDCIPP and DPHP to further explore these relationships. Although levels of BDCIPP and DPHP were on average higher for participants living in homes with the highest levels of TDCIPP and TPHP in dust (3rd tertile), effect estimates were imprecisely estimated and did not follow a consistent pattern across the exposure gradient (comparing the 3rd tertile to the 1st: 10^β^ = 1.27; 95% CI: 0.53, 3.04 and 10^β^ = 1.23; 95% CI: 0.57, 2.67; Table 5). Conversely, results suggest that categorical hand wipe levels of TDCIPP and TPHP may be associated with levels of BDCIPP and DPHP in participants’ urine (Table 5). Participants with the highest levels of TDCIPP on their hands, for instance, had urinary BDCIPP levels 1.99 times those of participants with the lowest levels of TDCIPP on their hands (95% CI: 0.89, 4.47).

**Table 4:** Correlation matrix for flame retardant levels measured in paired hand wipes (*n *= 53), dust (*n *= 49), and urine samples (*n *= 53).

		Dust		Hand wipes	
		TDCIPP	TPHP	TDCIPP	TPHP
Urine	BDCIPP	0.10	0.04	0.27	0.13
	DPHP	–0.17	0.15	0.17	0.37^#^
^#^< 0.01.					

**Table 5:** Regression analyses for predictors of urinary BDCIPP and DPHP.

Predictor	BDCIPP		DPHP	
	Coefficient^*a*^ (95% CI)	*p*-Value	Coefficient^*a*^ (95% CI)	*p*-Value
Sex
Male	Reference	—	Reference	—
Female	1.00 (0.51, 1.95)	0.99	1.84 (1.05, 3.21)	0.03
Age (years)	0.97 (0.94, 0.99)	0.008	0.98 (0.95, 1.00)	0.03
Visit time
Morning	Reference	—	Reference	—
Afternoon	2.15 (1.09, 4.27)	0.03	1.45 (0.78, 2.68)	0.23
Average times hands washed (times/day)
< 8	Reference	—	Reference	—
≥ 8	0.57 (0.28, 1.14)	0.11	0.90 (0.48, 1.68)	0.74
Hand sanitizer gel use
No	Reference	—	Reference	—
Yes	0.95 (0.46, 1.94)	0.89	0.74 (0.40, 1.38)	0.34
Average time active in the home (hr/day)
≤ 8	Reference	—	Reference	—
> 8	1.46 (0.68, 3.15)	0.16	1.23 (0.63, 2.42)	0.54
Average time driving in car (hr/day)
≤ 1	Reference	—	Reference	—
> 1	0.63 (0.32, 1.21)	0.16	0.81 (0.46, 1.46)	0.48
Dust TDCIPP or TPHP levels
Low	Reference	—	Reference	—
Mid	0.91 (0.38, 2.17)	0.67	0.86 (0.40, 1.87)	0.70
High	1.27 (0.53, 3.04)	0.72	1.23 (0.57, 2.67)	0.59
Hand wipe TDCIPP or TPHP congener levels
Low	Reference	—	Reference	—
Mid	1.51 (0.67, 3.39)	0.31	1.30 (0.66, 2.57)	0.44
High	1.99 (0.89, 4.47)	0.09	2.42 (1.23, 4.77)	0.01
^***a***^Exponentiated beta coefficients were used to represent the multiplicative change in urine concentrations relative to the reference group for categorical variables or the per-unit change for continuous variables (age).				

Several demographic and behavioral factors were also associated with the levels of PFR metabolites in urine samples. Women had significantly higher levels of DPHP in urine samples than men (10^β^ = 1.84; 95% CI: 1.05, 3.21; Table 5), and levels of both BDCIPP and DPHP decreased with age (10^β^ = 0.97; 95% CI: 0.94, 0.99 and 10^β^ = 0.98; 95% CI: 0.95, 1.00, respectively). Participants providing samples at the CRU in the afternoon tended to have higher levels of BDCIPP and DPHP in their urine than those who provided samples in the morning (10^β^ = 2.15; 95% CI: 1.09, 4.27 and 10^β^ = 1.45; 95% CI: 0.78, 2.68, respectively). Although not statically significant, results were suggestive of an inverse association between average hand washing frequency (< 8 times/day vs. ≥ 8 times/day) and the levels of BDCIPP and DPHP in urine (10^β^ = 0.57; 95% CI: 0.28, 1.14 and 10^β^ = 0.90; 95% CI: 0.48, 1.68, respectively).

*Temporal variation in urinary BDCIPP and DPHP*. For participants with repeated urine samples, the rank order of BDCIPP and DPHP urine concentrations was similar over time (see Supplemental Material, Figure S1). We examined the correlations between urine measures at each time point individually using Spearman correlations and found no evidence of reduced correlations over time (e.g., the correlation between each time point was similar; data not shown). Examining temporal variability in BDCIPP levels using ICCs, we observed strong consistency over the course of 5 consecutive days (ICC = 0.81; 95% CI: 0.75, 0.86) (Rosner 2000). DPHP levels in urine were also moderately to strongly consistent over the course of 5 days (ICC = 0.51; 95% CI: 0.42, 0.63).

## Discussion

Cumulatively, our results suggest that exposures to PFRs are common and vary in the general adult population. We found detectable levels of TDCIPP and TPHP in nearly all house dust and hand wipe samples. TDCIPP and TPHP were generally detected at levels well above those of BDE-47, BDE-99, BDE-100, BDE-153, and BDE-154. Levels of PFRs and PBDEs in dust were similar to those reported in recent studies in California and North Carolina (Dodson et al. 2012; Meeker et al. 2013; Stapleton et al. 2014). As products containing PBDEs are replaced with newer products containing alternative FRs, their levels may decrease. However, the levels of alternative FRs, such as TDCIPP and TPHP, may increase over time. Dodson et al. (2012), for example, reported declining levels of PBDEs in indoor dust collected in California homes (between 2006 and 2011), and increasing levels of alternative FRs, including TDCIPP, reflective of changes in FR applications in residential furniture (Stapleton et al. 2012b). Additional research is needed to determine whether the levels of TDCIPP and TPHP that we observed in the indoor environment and on participants’ hands affect human health.

The primary metabolites of TDCIPP and TPHP (i.e., BDCIPP and DPHP) were also detected in the vast majority of urine samples provided by study participants. Urinary DPHP and BDCIPP in our current work were approximately three times those reported previously in adult men (Meeker et al. 2013) similar to levels reported in office workers (Carignan et al. 2013) from the Boston, Massachusetts, area, and lower than in the levels we observed in a previous investigation of pregnant central North Carolina women (Hoffman et al. 2014). Near ubiquitous detection of PFR metabolites is of particular concern because the health impacts of PFR exposures remain largely unexplored in humans but *in vitro* and animal data suggest that they may be endocrine disruptors as well as carcinogenic (Babich 2006; Belcher et al. 2014; Farhat et al. 2013; Gold et al. 1978; Kojima et al. 2013; Liu et al. 2012, 2013; Wang et al. 2013).

Collecting paired house dust, hand wipe, and urine samples from study participants allowed us to examine associations between sample types and to explore potential pathways of exposure. We did not observe associations between measures of TDCIPP or TPHP in house dust and the levels on participants’ hands. There are several possible reasons for the lack of association. For example, hand wipe samples were collected at the CRU, but dust samples were collected in participants’ homes. It is possible that the levels of TDCIPP and TPHP on participants’ hands at the CRU were more reflective of recent TDCIPP and TPHP exposure, including exposure in other microenvironments that they may have recently visited (e.g., automobiles, the workplace, or the CRU). However, PBDEs in house dust were correlated with the levels on participants’ hands, which suggests that contact with PBDE-contaminated dust in the home environment was contributing to the levels of FRs on hand wipes, despite the measurements being taken at different times and locations (i.e., the home and the CRU). Differences in the physicochemical properties between PFRs and PBDEs may also explain these differences. TDCIPP, for example, is a smaller compound and has a higher vapor pressure than the PBDEs. Recent research from Weschler and Nazaroff (2012) speculated that semivolatile organic compounds in indoor air may sorb to skin, suggesting that the weaker association for the PFRs between hand wipes and dust may reflect a larger contribution of PFRs on hand wipes from the indoor air than from house dust. Similarly, Cao et al. (2014) recently demonstrated seasonal variation in the levels of PFRs in dust, but little variation in the levels of PBDEs.

Although dust samples were not associated with metabolites, higher levels of TDCIPP and TPHP on hand wipes were significantly associated with the levels of their metabolites in urine samples. Hand wipes may provide a more integrated picture of internal exposure, including information from multiple microenvironments, and may provide more biologically relevant measures of exposure than the levels of dust in a single room in the home. Although our work is the first to investigate relationships between dust and hand wipe PFRs with urinary metabolites, similar associations have been reported for PBDEs, with hand wipe levels being more strongly related to internal exposure than dust measures in a single microenvironment (e.g., homes or offices) (Stapleton et al. 2008, 2012a; Watkins et al. 2011). In addition, the strong relationship between the levels of TDCIPP and TPHP on hand wipes and the levels of their metabolites in urine suggests that hand-to-mouth contact or dermal absorption may be important pathways of exposure.

It is also interesting that DPHP concentrations in urine samples from women were almost twice those of men, which may suggest differences in exposure patterns by sex. For example, similar patterns have been observed for some phthalate metabolites (e.g., monobenzyl phthalate and monoethyl phthalate), a finding that has been attributed to differences in the use of personal care products between males and females (Silva et al. 2004). Alternatively, differences in the metabolism of TPHP between men and women may be driving the differences in the levels of DPHP in urine. Although TPHP is reportedly used in nail polish, we are not aware of other common personal care products in which it is used. In addition, we observed higher levels of BDCIPP and DPHP for participants who provided urine samples in the afternoon, suggesting differences in exposure patterns throughout the day.

*In vivo* and *in vitro* studies suggest that TDCIPP and TPHP are rapidly metabolized (to BDCIPP and DPHP, respectively) and eliminated from the body (Cooper et al. 2011; Lynn et al. 1981; Nomeir et al. 1981). We observed moderate to strong reliability in the levels of BDCIPP and DPHP in urine samples collected on 5 consecutive days. The observed ICCs (0.81 for BDCIPP and 0.51 for DPHP) were much greater than those typically reported for rapidly metabolized compounds with primarily dietary sources [e.g., organophosphate pesticides (Bradman et al. 2013)]. Previous studies that assessed the reliability of repeated measures to BDCIPP and DPHP in pregnant women and in adult men have also reported moderate to strong reliability [for three measurements throughout pregnancy, DPHP ICC = 0.5 and BDCIPP ICC = 0.6 (Hoffman et al. 2014); for nine samples over 3 months, DPHP ICC = 0.7 and BDCIPP ICC = 0.5 (Meeker et al. 2013)]. These findings suggest that TDCIPP and TPHP may come from more continuous sources of exposure, such as contact with products containing PFRs or contact with contaminated dust. Nonetheless, variation in daily behavior (e.g., working in an office environment or spending more time at home) may affect levels of exposure to PFRs.

Our study has several limitations that should be considered in the interpretation of results. First, paired dust, hand wipe, and urine samples were each collected only once; multiple samples taken over time and in different microenvironments (e.g., workplaces and cars) may provide additional insights to important routes of exposure to PFRs. Second, we did not measure the concentrations of TDCIPP or TPHP in indoor air. Both TDCIPP and TPHP have been detected in household air samples previously (Staaf and Ostman 2005), and data suggest that inhalation exposure may be an important pathway to consider in future assessments (Stapleton et al. 2009). Third, although detailed instructions were provided, household dust samples were collected by participants; variability in the areas sample and the types of vacuums used may have introduced measurement error into our analyses. We expect that any measurement error introduced by differences in dust collection between participants was not related to the levels of TDCIPP or TPHP in house dust and, therefore, may have biased our result toward the null. Fourth, our small sample size limited the number of predictive variables that we could include in multivariate regression analyses at the same time and may have limited our power to detect meaningful associations. Finally, although participants were recruited from the general North Carolina population, the cohort was a relatively homogeneous group; participants were primarily white and there was little variability in behavioral characteristics. Although this may limit our ability to generalize results to the broader U.S. population, it does not alter the internal validity of our results.

## Conclusions

Cumulatively, our results indicate that PFR exposures are widespread in the general adult population. Hand-to-mouth contact or dermal absorption may be important pathways of exposure because the levels of TDCIPP and TPHP on hand wipes were associated with the levels of their metabolites in urine. Our results suggest that hand wipe measures of TDCIPP and TPHP may provide a means of characterizing exposure to PFRs in future epidemiologic studies. Such studies are needed to determine whether the levels of TDCIPP and TPHP that we observed in the indoor environment impact human health, particularly because animal studies suggest that PFRs may adversely affect health.

## Supplemental Material

(519 KB) PDF
