# Supplementary material for: Monitoring Indoor Exposure to Organophosphate Flame Retardants: Hand Wipes and House Dust
Source: Environ Health Perspect. 2014 Oct 24;123(2):160–5. doi: 10.1289/ehp.1408669 (PMC4314253; doi:10.1289/ehp.1408669)

Environmental Health Perspectives doi:10.1289/ehp.1408669

Supplemental Material

Monitoring Indoor Exposure to Organophosphate Flame Retardants: Hand Wipes and House Dust

Kate Hoffman, Stavros Garantziotis, Linda S. Birnbaum, and Heather M. Stapleton

Table S1. Regression analyses [coefficient[a] (95% CI)] for predictors of handwipe flame retardant levels. Analyses were conducted on dust and handwipe data in which detection frequency was >70%.

Predictor	TDCIPP	TPHP	BDE47	BDE99	BDE100	BDE153	BDE154	BDE209
Sex								
Male	Reference	Reference	Reference	Reference	Reference	Reference	Reference	Reference
Female	0.95 (0.55, 1.62)	1.56 (0.83, 2.93)	0.85 (0.42, 1.72)	1.05 (0.54, 2.02)	0.85 (0.38, 1.87)	1.03 (0.43, 2.47)	0.81 (0.34, 1.91)	0.53 (0.25, 1.09)
Age (years)	0.98 (0.96, 1.00)	0.99 (0.97, 1.02)	1.01 (0.98, 1.04)	1.01 (0.98, 1.04)	1.01 (0.97, 1.04)	1.01 (0.98, 1.05)	1.01 (0.98, 1.05)	1.02 (0.99, 1.05)
Visit time								
Morning	Reference	Reference	Reference	Reference	Reference	Reference	Reference	Reference
Afternoon	1.06 (0.60, 1.87)	1.14 (0.58, 2.25)	1.07 (0.51, 2.28)	1.05 (0.52, 2.12)	0.84 (0.36, 1.95)	1.27 (0.50, 3.22)	1.44 (0.58, 3.59)	1.13 (0.50, 2.52)
Average times hands washed per day								
<8 times/day	Reference	Reference	Reference	Reference	Reference	Reference	Reference	Reference
≥8 times/day	0.76 (0.43, 1.33)	1.24 (0.63, 2.47)	0.61 (0.29, 1.28)	0.66 (0.33, 1.32)	0.57 (0.25, 1.32)	0.46 (0.19, 1.15)	0.41 (0.17, 0.99)	1.22 (0.55, 2.73)
Handgel use								
No	Reference	Reference	Reference	Reference	Reference	Reference	Reference	Reference
Yes	0.74 (0.42, 1.30)	0.66 (0.33, 1.29)	0.79 (0.37, 1.69)	0.90 (0.44, 1.81)	1.08 (0.46, 2.53)	0.80 (0.31, 2.04)	0.89 (0.35, 2.23)	0.82 (0.37, 1.82)
Average time active in the home per day								
≤8 hours/day	Reference	Reference	Reference	Reference	Reference	Reference	Reference	Reference
>8 hours/day	1.34 (0.73, 2.49)	0.77 (0.37, 1.62)	1.12 (0.50, 2.54)	1.32 (0.62, 2.83)	0.84 (0.33, 2.10)	1.92 (0.71, 5.22)	1.59 (0.59, 4.28)	1.53 (0.64, 3.63)
Average time driving in car per day								
≤1 hour/day	Reference	Reference	Reference	Reference	Reference	Reference	Reference	Reference
>1 hour/day	0.81 (0.48, 1.38)	1.33 (0.70, 2.53)	1.21 (0.60, 2.44)	1.29 (0.67, 2.48)	0.9 (0.41, 2.00)	0.83 (0.35, 2.00)	1.11 (0.47, 2.63)	1.88 (0.91, 3.91)
Dust congener levels								
Low	Reference	Reference	Reference	Reference	Reference	Reference	Reference	Reference
Mid	0.90 (0.45, 1.84)	1.20 (0.51, 2.82)	1.36 (0.55, 3.37)	1.29 (0.56, 2.97)	1.61 (0.59, 4.43)	2.94 (0.99, 8.75)	2.16 (0.73, 6.41)	2.34 (0.90, 6.06)
High	1.18 (0.59, 2.39)	1.08 (0.46, 2.54)	2.62 (1.05, 6.49)	2.45 (1.06, 5.66)	3.44 (1.25, 9.44)	5.13 (1.73, 15.22)	3.49 (1.18, 10.35)	2.32 (0.89, 6.01)

[a]Exponentiated beta-coefficients represent the multiplicative change in urine concentrations relative to the reference group for categorical variables, or the per unit change for continuous variables (age and BMI).

Figure S1. Concentrations (ng/mL) of metabolites in urine from repeated samples collected over five consecutive days. (A) BDCIPP; (B) DPHP. Individual participants are shown using different symbols and colors. Measured samples are connected to assist in the interpretation of changes over time. Dashed lines indicate that information is missing at a sample time point. Measurements are aligned by day of the week.

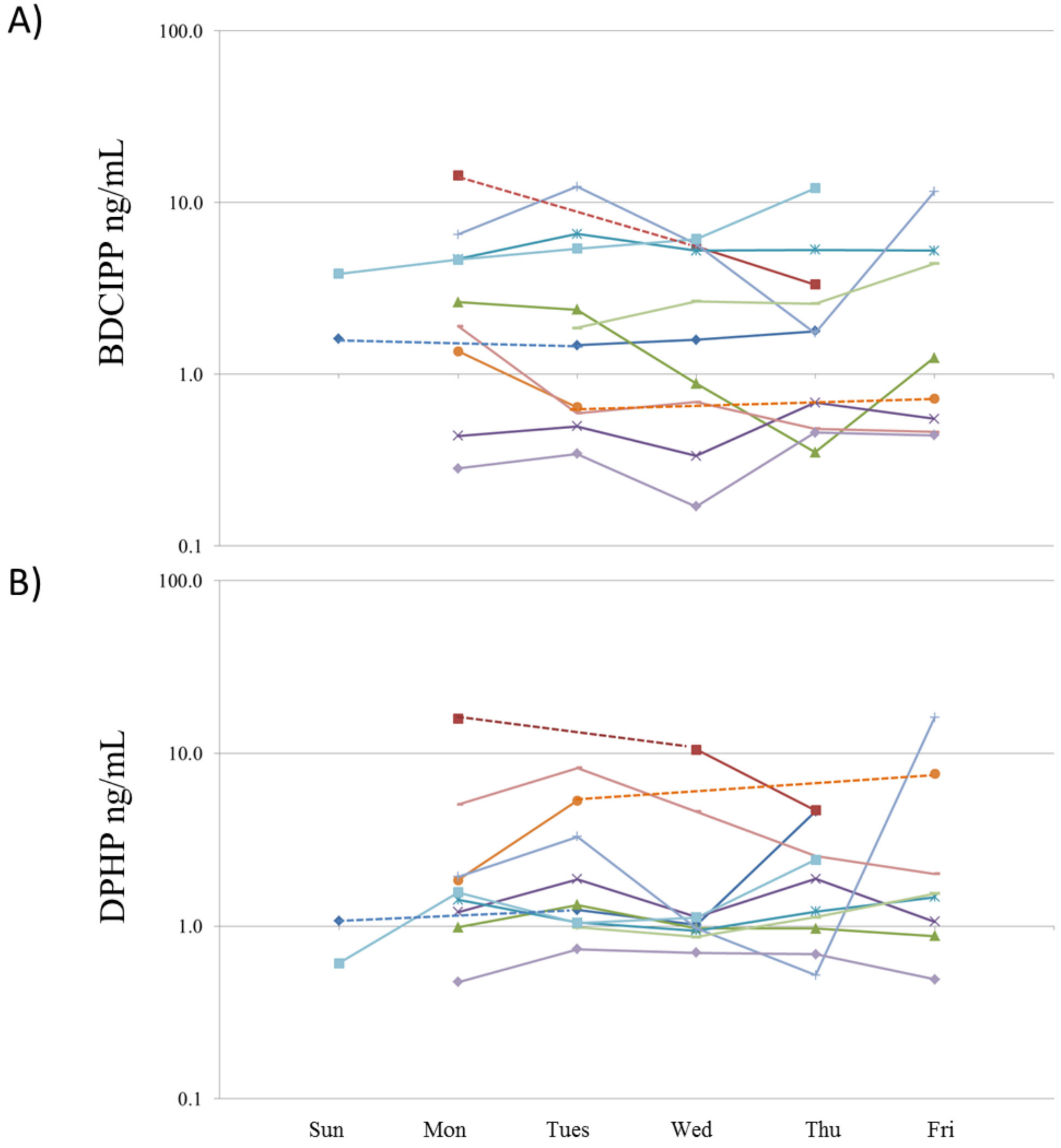

Supplement: (519 KB) PDF [file ehp.1408669.s001.508.pdf]
